# Association of the CUN-BAE body adiposity estimator and other obesity indicators with cardiometabolic multimorbidity: a cross-sectional study

**DOI:** 10.1038/s41598-024-52164-7

**Published:** 2024-05-08

**Authors:** Xuejiao Chen, Shuoji Geng, Zhan Shi, Jiacheng Ding, Haojie Li, Donghai Su, Yulin Cheng, Songhe Shi, Qingfeng Tian

**Affiliations:** 1 Department of Epidemiology and Health Statistics, College of Public Health, Zhengzhou University, 100 Kexue Avenue, Zhengzhou, 450001 Henan People’s Republic of China; 2 Department of Pharmacy, Zhengzhou People’s Hospital, Zhengzhou, Henan People’s Republic of China; 3 Department of Social Medicine and Health Management, College of Public Health, Zhengzhou University, Zhengzhou, China

**Keywords:** Diseases, Medical research, Risk factors

## Abstract

Cardiometabolic multimorbidity (CM), defined as the coexistence of two or three cardiometabolic disorders, is one of the most common and deleterious multimorbidities. This study aimed to investigate the association of Clínica Universidad de Navarra-Body Adiposity Estimator (CUN-BAE), body mass index (BMI), waist circumference (WC), and waist-to-height ratio (WHtR) with the prevalence of CM. The data were obtained from the 2021 health checkup database for residents of the Electronic Health Management Center in Xinzheng, Henan Province, China. 81,532 participants aged ≥ 60 years were included in this study. Logistic regression models were used to estimate the odd ratios (ORs) and 95% confidence intervals (CIs) for CUN-BAE, BMI, WC, and WHtR in CM. The area under the receiver operating characteristic curve (AUC) was used to compare the discriminatory ability of different anthropometric indicators for CM. The multivariable-adjusted ORs (95% CIs) (per 1 SD increase) of CM were 1.799 (1.710–1.893) for CUN-BAE, 1.329 (1.295–1.364) for BMI, 1.343 (1.308–1.378) for WC, and 1.314 (1.280–1.349) for WHtR, respectively. Compared with BMI, WC and WHtR, CUN-BAE had the highest AUC in both males and females (AUC: 0.642; 95% CI 0.630–0.653 for males, AUC: 0.614; 95% CI 0.630–0.653 for females). CUN-BAE may be a better measure of the adverse effect of adiposity on the prevalence of CM than BMI, WC, and WHtR.

## Introduction

Multimorbidity is the coexistence of at least two chronic conditions in a person and is associated with reduced quality of life and greater use of health resources^1–3^. With rapid population aging and urbanization, it is becoming a global public health challenge. Cardiometabolic multimorbidity (CM) is one of the most common and harmful multimorbidity profiles and is defined as the coexistence of two or three cardiometabolic diseases, including diabetes, coronary heart disease, and stroke^4–7^. Remarkably, the health damage caused by CM is much greater than that caused by a single cardiometabolic disease. It is reported that older adults with CM have about 15 years less life expectancy, which is almost twice as long as any single cardiometabolic disease^8^. In addition, participants with single cardiometabolic disease and CM had 1.4 times and 1.9 times the risk of mental stress compared to those without cardiometabolic disease^9^. However, existing studies mainly focus on single cardiometabolic disease, while CM, a growing public health problem, is rather understudied, and early preventive measures are urgently needed.

Among several modifiable risk factors for cardiometabolic disease, obesity is considered to be a major risk factor^10^. In the general population, the most widely used measures to define excess body fatness (BF) are body mass index (BMI), waist circumference (WC), and waist-to-height ratio (WHtR). Previous studies have shown that higher BMI, WC, and WHtR are associated with an increased risk of CM^4,11^. However, these anthropometric indicators have been criticized because they do not take into account important factors related to adiposity, especially age, gender, or race^12,13^. Therefore, a new practical adiposity index, the Clínica Universidad de Navarra-Body Adiposity Estimator (CUN-BAE) was proposed and considered to be a more accurate indicator of body fat estimation^14^. This method of estimating body fat percentage was based on body mass index, sex, and age in 6510 Caucasian subjects, with validation using air displacement plethysmography (ADP) as the gold standard, which has the highest correlation with body fat percent as measured by air displacement volume tracing compared to other anthropometric methods. Because of the accuracy of CUN-BAE in predicting BF% and its correlation with CVD and metabolic conditions, it has been widely used in clinical studies^15–19^. Previous findings have shown that CUN-BAE is more strongly associated with fat-related cardiovascular risk factors than BMI or WC^18,19^. However, the relationship between CUN-BAE, BMI, WC, WHtR, and CM has not been evaluated.

Therefore, the purpose of this study was to investigate the association of CUN-BAE, BMI, WC, and WHtR with the prevalence of CM in the Chinese elderly population and to compare the predictive ability of CUN-BAE, BMI, WC, and WHtR on the prevalence of CM.

## Methods

### Study design and participants

This study is a cross-sectional study based on a large sample of people aged 60 years and older. The data were obtained from the 2021 resident health checkup database of the Electronic Health Management Center in Xinzheng, Henan Province, Central China. Xinzheng's health check-up program for residents is an important part of China's basic public health service program. From January to December 2021, 117,517 participants were eligible for the study. We excluded participants with the following conditions: (1) participants aged < 60 years (n = 35,062); (2) missing information for marriage, drinking, smoking, exercise, height, weight, WC, resting heart rate (RHR), systolic blood pressure (SBP) or diastolic blood pressure (DBP) (n = 923). Finally, the study included 81,532 participants. The data screening flow chart is presented in Supplementary Fig. S1. This study was approved by the Ethics Committee of Zhengzhou University (Reference Number: ZZUIRB2019-019), and informed consent was obtained from all participants. All methods were carried out followed the relevant guidelines and regulations.

### Data collection

Demographic and clinical information was collected from participants at the health screening. Demographic information included sex, age, marital status, smoking, drinking, and physical activity. Marital status was categorized into couples and singles, with singles including unmarried, divorced, and widowed. Smoking was defined as never smokers, former smokers, and current smokers. Drinking was classified as never, occasionally, and daily. Physical exercise was divided into four categories: never, occasionally, more than once a week and daily. Clinical data included anthropometric measurements, laboratory investigations, and self-reported disease history. The participants of the study wore light clothing and bare feet for height, weight, and waist measurements. Body height and weight were measured via a standard digital weighing scale and stadiometer, respectively. WC was measured using a calibrated tape measure while the subject was standing and during slight expiration. WHtR was calculated as WC (m)/height (m). CUN-BAE = − 44.988 + (0.503 × age) + (10.689 × sex) + (3.172 × BMI) − (0.026 × BMI^2^) + (0.181 × BMI × sex)− (0.02 × BMI × age) − (0.005 × BMI^2^ × sex) + (0.00021 × BMI^2^ × age) where male = 0 and female = 1 for sex, and age in years^14^. Resting heart rate (RHR), SBP, and DBP were measured twice using an automatic sphygmomanometer (Omron HEM-7125, Kyoto, Japan)^20^ after the subjects rested in a seated position for at least 5 min, and the mean value was recorded as the final results.

### Definition of CM

CM is defined as having two or more of the following three diseases: diabetes, stroke, and coronary heart disease^4–7^. The diagnosis of cardiometabolic disease is determined by physician diagnosis or self-reported information from the participant.

### Statistical analysis

Continuous variables were expressed as means and standard deviations (SDs). Categorical variables were expressed as numbers and frequencies. The chi-square test for categorical variables and the Kruskal–Wallis test for continuous variables were used to compare the differences between the two groups defined by the CM. The associations of CUN-BAE, BMI, WC, and WHtR with CM were analyzed by logistic regression models, and ORs with 95% CIs of CUN-BAE, BMI, WC, and WHtR in quartiles and continuous variables were expressed in separate models. Model 1 was unadjusted. Model 2 adjusted for sex, age, and marital status. Model 3 adjusted for confounders including sex, age, marital status, smoking, drinking, physical exercise, SBP, DBP, and RHR. The dose–response association and the potentially nonlinear relationship of continuous CUN-BAE, BMI, WC, and WHtR with CM were explored by restricted cubic spline models with four knots. In addition, stratified analysis was performed by subgroups of age and sex using a logistic regression model to test the consistency of these relationships. The interaction of four obesity indicators with sex and age was assessed. Finally, the receiver operating characteristics (ROC) curve and related area under the ROC curve (AUC) were used to compare the capability of CUN-BAE, BMI, WC, and WHtR to diagnose CM. The statistical analyses were performed using SPSS V 21 and R V 4.0.3. *P* < 0.05 for a two-sided test was considered statistically significant.

### Ethics approval

The study was approved by the Ethics Committee of Zhengzhou University (Reference Number: ZZUIRB2019-019), and written informed consent was obtained from all participants.

## Results

### Characteristics of the study population

The baseline characteristics of the study subjects with and without CM are presented in Table 1. Overall, 81,532 subjects were studied, 53.1% of whom were women and 46.9% of whom were men. A total of 5,767 participants had CM, and the prevalence rate was 7.1%. Subjects who developed CM had higher levels of CUN-BAE, BMI, WC, and WHtR than those who did not (*P* < 0.001). The correlations between CUN-BAE, BMI, WC, and WHtR are shown in Table S1.

**Table 1:** Baseline characteristics of the study population with and without CM.

Characteristics	Total (n = 81,532)	Noncardiometabolic multimorbidity (n = 75,765)	Cardiometabolic multimorbidity (n = 5767)	*P* value
Age (years)				< 0.001
Younger elderly (60–74)	62,203 (76.29)	58,178 (76.79)	4025 (69.79)	
Older adults (≥ 75)	19,329 (23.71)	17,587 (23.21 )	1742 (30.21)	
Gender (%)				< 0.001
Men	38,241 (46.90)	36,023 (47.55)	2218 (38.46)	
Women	43,291 (53.10)	39,742 (52.45)	3549 (61.54)	
Marital status				0.093
Single	12,091 (14.83)	11,192 (14.77)	899 (15.59)	
Couple	69,441 (85.17)	64,573 (85.23)	4868 (84.41)	
Physical exercise (%)				< 0.001
Never	54,120 (66.39)	50,579 (66.76)	3541 (61.40)	
Occasionally	1469 (1.80)	1358 (1.79)	111 (1.92)	
More than once a week	3268 (4.00)	3027 (4.00)	241 (4.18)	
Daily	22,675 (27.81)	20,801 (27.45)	1874 (32.50)	
Smoking (%)				< 0.001
Never smokers	71,516 (87.72)	66,305 (87.51)	5211 (90.36)	
Former smokers	1291 (1.58)	1169 (1.54)	122 (2.12)	
Current smokers	8725 (10.70)	8291 (10.94)	434 (7.53)	
Drinking (%)				0.001
Never	76,870 (94.28)	71,378 (94.21)	5492 (95.23)	
Occasionally	2634 (3.23)	2461 (3.25)	173 (3.00)	
Daily	2028 (2.49)	1926 (2.54)	102 (1.77)	
RHR	71.5 ± 11.6	71.4 ± 11.5	73.5 ± 13.0	< 0.001
SBP (mmHg)	145.5 ± 20.2	145.2 ± 20.2	149.4 ± 20.5	< 0.001
DBP (mmHg)	84.0 ± 10.7	83.9 ± 10.7	84.7 ± 11.2	< 0.001
BMI (kg/m^2^)	25.55 ± 3.59	25.47 ± 3.57	26.61 ± 3.69	< 0.001
WC	86.88 ± 9.26	86.68 ± 9.19	89.50 ± 9.71	< 0.001
WHtR	0.55 ± 0.06	0.55 ± 0.06	0.57 ± 0.06	< 0.001
CUN-BAE	34.37 ± 7.49	34.19 ± 7.48	36.74 ± 7.17	< 0.001

Data are presented as means ± SD or number (percentage).

*BMI* body mass index, *WC* waist circumference, *WHtR* waist-to-height ratio, *CUN-BAE* Clínica Universidad de Navarra-Body Adiposity Estimator, *SBP* systolic blood pressure, *DBP* diastolic blood pressure; RHR, resting heart rate.

### Prevalence of CM by CUN-BAE, BMI, WC, and WHtR

Table 2 presents the ORs and 95% CIs for the association of CM with the four indicators (CUN-BAE, BMI, WC, and WHtR) in the general population. In this study, CUN-BAE, BMI, WC, and WHtR were all positively associated with the prevalence of CM in a dose–response relationship (*P* trend < 0.001). In the total population, after adjusting for other covariates including age, sex, marital status, drinking, smoking, physical activity, SBP, DBP, and RHR, in Model 3, the OR (95% CI) for CM with per SD increase in CUN-BAE, BMI, WC, and WHtR were 1.799 (1.710–1.893), 1.329 (1.295–1.364), 1.343 (1.308–1.378), and 1.314 (1.280–1.349), respectively, and the cumulative risk of CM increased with by CUN-BAE, BMI, WC, and WHtR quartile (OR (95% CI): 1.823 (1.665–1.995), 2.570 (2.234–2.956), and 4.057 (3.505–4.695) for CUN-BAE, 1.337 (1.225–1.459), 1.682 (1.545–1.831), and 2.251 (2.075–2.442) for BMI, 1.114 (1.019–1.218), 1.496 (1.376–1.626), and 2.072 (1.915–2.242) for WC, and 1.319 (1.202–1.447), 1.592 (1.452–1.745), and 2.214 (2.027–2.418) for WHtR, for quartiles 2, 3, and 4 versus quartile 1, respectively).

**Table 2:** Association between BMI, WC, WHtR, CUN-BAE, and CM.

Variables	No. of cases	Model 1 OR (95% CI)	Model 2 OR (95% CI)	Model 3 OR (95% CI)
BMI				
< P^25^	949	Reference	Reference	Reference
P^25^-P^50^	1233	1.305 (1.196–1.423)	1.349 (1.236–1.472)	1.337 (1.225–1.459)
P^50^-P^75^	1528	1.653 (1.520–1.797)	1.724 (1.585–1.875)	1.682 (1.545–1.831)
≥ P^75^	2057	2.283 (2.109–2.472)	2.354 (2.172–2.551)	2.251 (2.075–2.442)
P trend		< 0.001	< 0.001	< 0.001
Continuous per SD	5767	1.348 (1.314–1.382)	1.352 (1.318–1.387)	1.329 (1.295–1.364)
WC				
< P^25^	1015	Reference	Reference	Reference
P^25^-P^50^	1062	1.088 (0.996–1.189)	1.138 (1.042–1.244)	1.114 (1.019–1.218)
P^50^-P^75^	1497	1.440 (1.326–1.563)	1.556 (1.432–1.690)	1.496 (1.376–1.626)
≥ P^75^	2193	2.010 (1.861–2.171)	2.191 (2.027–2.368)	2.072 (1.915–2.242)
P trend		< 0.001	< 0.001	< 0.001
Continuous per SD	5767	1.335 (1.301–1.369)	1.368 (1.334–1.403)	1.343 (1.308–1.378)
WHtR				
< P^25^	747	Reference	Reference	Reference
P^25^-P^50^	1308	1.387 (1.265–1.520)	1.363 (1.242–1.494)	1.319 (1.202–1.447)
P^50^-P^75^	1450	1.757 (1.605–1.924)	1.679 (1.532–1.839)	1.592 (1.452–1.745)
≥ P^75^	2262	2.582 (2.371–2.812)	2.387 (2.188–2.604)	2.214 (2.027–2.418)
P trend		< 0.001	< 0.001	< 0.001
Continuous per SD	5767	1.387 (1.353–1.422)	1.346 (1.312–1.381)	1.314 (1.280–1.349)
CUN-BAE				
< P^25^	821	Reference	Reference	Reference
P^25^-P^50^	1425	1.789 (1.638–1.954)	1.898 (1.735–2.076)	1.823 (1.665–1.995)
P^50^-P^75^	1399	1.756 (1.608–1.919)	2.723 (2.370–3.130)	2.570 (2.234–2.956)
≥ P^75^	2122	2.761 (2.541–3.000)	4.406 (3.813–5.092)	4.057 (3.505–4.695)
P trend		< 0.001	< 0.001	< 0.001
Continuous per SD	5767	1.418 (1.379–1.458)	1.863 (1.773–1.959)	1.799 (1.710–1.893)

*OR* odd ratio, *CI* confidential interval, *BMI* body mass index, *WC* waist circumference, *WHtR* waist-to-height ratio, *CUN-BAE* Clínica Universidad de Navarra-Body Adiposity Estimator, *SBP* systolic blood pressure, *DBP* diastolic blood pressure, *RHR* resting heart rate.

Model 1: unadjusted.

Model 2: adjusted for age, sex, and marital status.

Model 3: Model 2 plus smoking, drinking, physical activity, SBP, DBP, and RHR.

Table 3 presents a stratified analysis by gender group and shows that higher CUN-BAE, BMI, WC, and WHtR were associated with a higher prevalence of CM in both men and women. The same trend was observed in the subgroup analysis stratified by age in Table S2. The results of the interaction of CUN-BAE, BMI, WC, and WHtR with sex and age are shown in Table S3.

**Table 3:** Association between BMI, WC, WHtR, CUN-BAE, and CM by different sex.

Variables	No. of cases	Model 1 OR (95% CI)	Model 2 OR (95% CI)	Model 3 OR (95% CI)
Male				
BMI				
< P^25^	345	Reference	Reference	Reference
P^25^-P^50^	448	1.315 (1.140–1.518)	1.375 (1.191–1.588)	1.355 (1.173–1.566)
P^50^-P^75^	605	1.796 (1.569–2.057)	1.905 (1.662–2.183)	1.834 (1.598–2.105)
≥ P^75^	820	2.502 (2.199–2.847)	2.682 (2.354–3.055)	2.523 (2.209–2.881)
P trend		< 0.001	< 0.001	< 0.001
Continuous per SD	2218	1.397 (1.341–1.455)	1.425 (1.368–1.484)	1.394 (1.337–1.454)
WC				
< P^25^	291	Reference	Reference	Reference
P^25^-P^50^	431	1.238 (1.063–1.440)	1.257 (1.080–1.463)	1.228 (1.054–1.430)
P^50^-P^75^	594	1.761 (1.526–2.033)	1.821 (1.577–2.103)	1.746 (1.510–2.018)
≥ P^75^	902	2.621 (2.289–3.001)	2.707 (2.362–3.101)	2.534 (2.207–2.909)
P trend		< 0.001	< 0.001	< 0.001
Continuous per SD	2218	1.434 (1.378–1.493)	1.446 (1.389–1.505)	1.415 (1.358–1.474)
WHtR				
< P^25^	303	Reference	Reference	Reference
P^25^-P^50^	391	1.196 (1.026–1.393)	1.190 (1.021–1.387)	1.149 (0.985–1.340)
P^50^-P^75^	611	1.535 (1.334–1.768)	1.520 (1.320–1.750)	1.440 (1.249–1.660)
≥ P^75^	913	2.457 (2.151–2.808)	2.414 (2.112–2.759)	2.220 (1.938–2.543)
P trend		< 0.001	< 0.001	< 0.001
Continuous per SD	2218	1.396 (1.342–1.453)	1.384 (1.330–1.440)	1.349 (1.295–1.405)
CUN-BAE				
< P^25^	305	Reference	Reference	Reference
P^25^-P^50^	439	1.458 (1.256–1.692)	1.392 (1.199–1.616)	1.366 (1.175–1.587)
P^50^-P^75^	637	2.163 (1.881–2.487)	2.020 (1.756–2.325)	1.952 (1.694–2.250)
≥ P^75^	837	2.900 (2.536–3.317)	2.684 (2.344–3.074)	2.524 (2.198–2.897)
P trend		< 0.001	< 0.001	< 0.001
Continuous per SD	2218	1.484 (1.422–1.548)	1.450 (1.389–1.514)	1.418 (1.356–1.481)
Female				
BMI				
< P^25^	610	Reference	Reference	Reference
P^25^-P^50^	790	1.314 (1.178–1.465)	1.388 (1.244–1.549)	1.373 (1.230–1.533)
P^50^-P^75^	949	1.603 (1.442–1.781)	1.723 (1.549–1.917)	1.683 (1.512–1.874)
≥ P^75^	1200	2.078 (1.877–2.299)	2.259 (2.038–2.504)	2.174 (1.959–2.412)
P trend		< 0.001	< 0.001	< 0.001
Continuous per SD	3549	1.296 (1.255–1.339)	1.328 (1.285–1.373)	1.309 (1.266–1.353)
WC				
< P^25^	545	Reference	Reference	Reference
P^25^-P^50^	745	1.139 (1.016–1.277)	1.155 (1.031–1.295)	1.136 (1.013–1.274)
P^50^-P^75^	972	1.466 (1.315–1.635)	1.509 (1.353–1.683)	1.453 (1.303–1.622)
≥ P^75^	1287	2.017 (1.817–2.238)	2.080 (1.873–2.309)	1.973 (1.775–2.194)
P trend		< 0.001	< 0.001	< 0.001
Continuous per SD	3549	1.311 (1.269–1.355)	1.324 (1.281–1.368)	1.300 (1.257–1.344)
WHtR				
< P^25^	460	Reference	Reference	Reference
P^25^-P^50^	796	1.341 (1.191–1.509)	1.344 (1.193–1.513)	1.302 (1.156–1.466)
P^50^-P^75^	918	1.682 (1.498–1.888)	1.669 (1.486–1.874)	1.591 (1.416–1.788)
≥ P^75^	1,375	2.180 (1.954–2.432)	2.145 (1.923–2.394)	2.004 (1.794–2.239)
P trend		< 0.001	< 0.001	< 0.001
Continuous per SD	3549	1.319 (1.276–1.363)	1.309 (1.267–1.353)	1.281 (1.239–1.325)
CUN-BAE				
< P^25^	554	Reference	Reference	Reference
P^25^-P^50^	767	1.406 (1.256–1.574)	1.365 (1.219–1.529)	1.351 (1.207–1.513)
P^50^-P^75^	999	1.875 (1.684–2.088)	1.805 (1.620–2.011)	1.766 (1.584–1.969)
≥ P^75^	1229	2.363 (2.129–2.622)	2.272 (2.047–2.523)	2.188 (1.969–2.432)
P trend		< 0.001	< 0.001	< 0.001
Continuous per SD	3549	1.364 (1.318–1.411)	1.347 (1.302–1.395)	1.327 (1.281–1.375)

*OR* odd ratio, *CI* confidential interval, *BMI* body mass index, *WC* waist circumference, *WHtR* waist-to-height ratio, *CUN-BAE* Clínica Universidad de Navarra-Body Adiposity Estimator, *SBP* systolic blood pressure, *DBP* diastolic blood pressure, *RHR* resting heart rate.

Model 1: unadjusted.

Model 2: adjusted for age, sex, and marital status.

Model 3: Model 2 plus smoking, drinking, physical activity, SBP, DBP, and RHR.

### Restricted cubic spline curves for four indicators and CM

**Figure 1:**
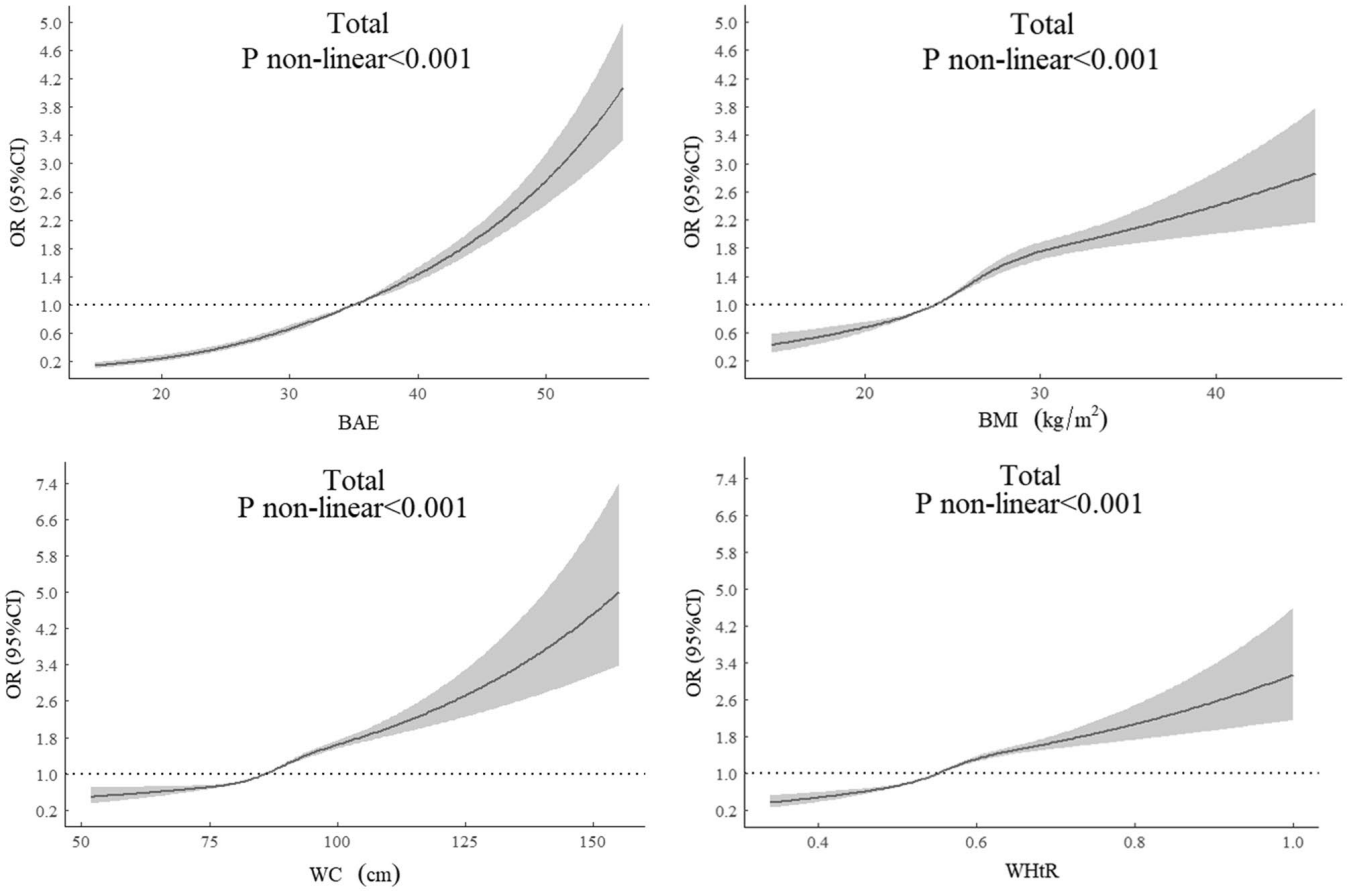
Odd ratios for the association between CUN-BAE, BMI, WC, and WHtR and CM risk in all participants. ORs are adjusted for age, sex, Marital status, drinking, smoking, physical activity, SBP, DBP, and RHR. OR, odd ratio; SBP, systolic blood pressure; DBP, diastolic blood pressure; RHR, resting heart rate; CUN-BAE, Clínica Universidad de Navarra-Body Adiposity Estimator; BMI, body mass index; WC, waist circumference; WHtR, waist-to-height ratio.

Multivariable adjusted restricted cubic spline analysis showed the dose–response relationship between CUN-BAE, BMI, WC, WHtR, and CM for all participants in Fig. 1, and the results showed that the prevalence of CM increased with increasing CUN-BAE, BMI, WC, and WHtR. The associations of CUN-BAE, BMI, WC, WHtR, and CM were nonlinear in all participants.

### The receiver operating characteristic curves for four indicators and CM.

**Figure 2:**
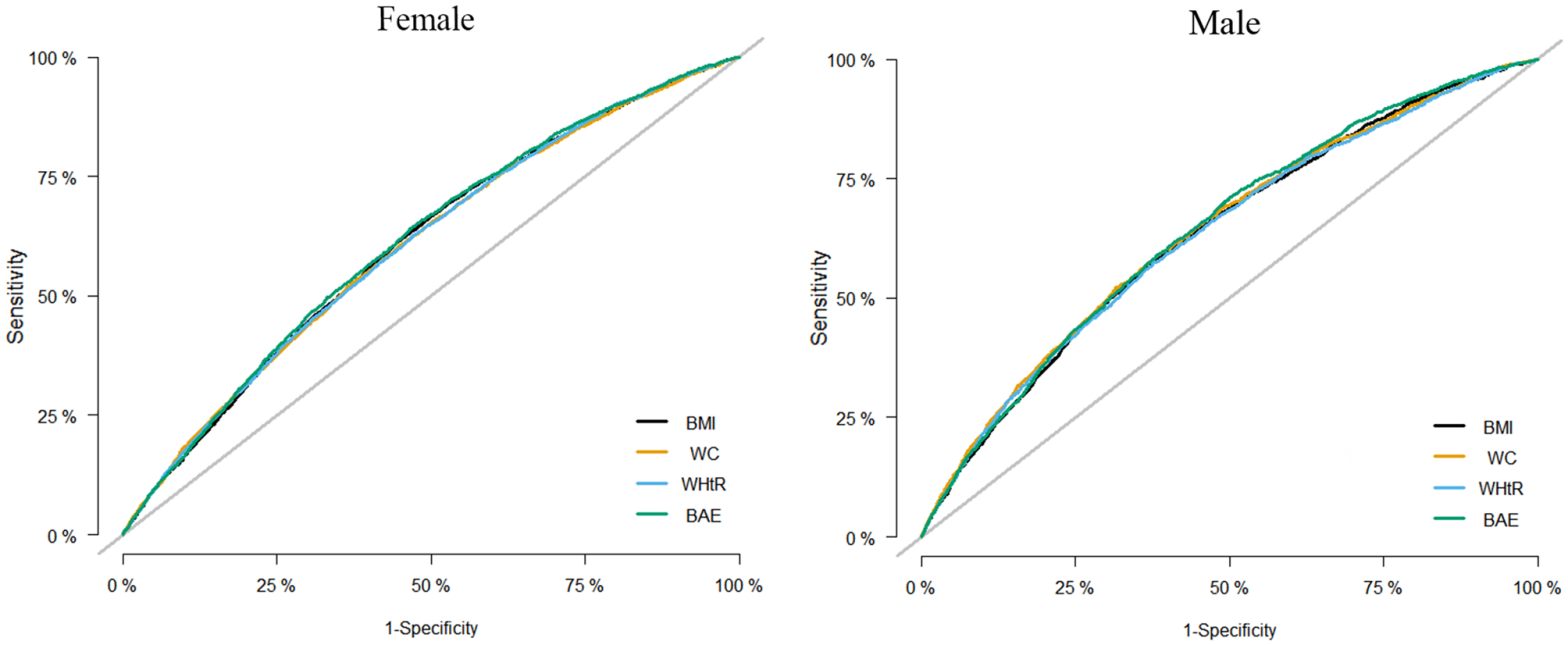
The receiver operating characteristic curve of anthropometric indicators after adjusting for age, sex, Marital status, drinking, smoking, physical activity, SBP, DBP, and RHR. OR, odd ratio; SBP, systolic blood pressure; DBP, diastolic blood pressure; RHR, resting heart rate; CUN-BAE, Clínica Universidad de Navarra-Body Adiposity Estimator; BMI, body mass index; WC, waist circumference; WHtR, waist-to-height ratio.

As shown in Table 4, the AUCs of CUN-BAE, BMI, WC, and WHtR for CM were calculated after adjusting for sex, age, marital status, smoking, drinking, physical activity, SBP, DBP, and RHR to compare the ability of these indicators to identify CM. The ROCs are shown in Fig. 2. The best indicator for identifying CM in both males and females was CUN-BAE (AUC: 0.642; 95% CI 0.630 to 0.653 for males, AUC: 0.614; 95% CI 0.630 to 0.653 for females). The AUCs of CUN-BAE, BMI, WC, and WHtR for diabetes, stroke, and coronary heart disease after adjusting for a range of confounders were shown in Table S4. In females, CUN-BAE is the best indicator for the identification of stroke and coronary heart disease, and in the identification of diabetes, CUN-BAE has the same validity as BMI. Among males, BMI, CUN-BAE, and WHtR were the best indicators to identify diabetes, stroke, and coronary heart disease, respectively.

**Table 4:** AUCs for anthropometric indices to CM.

Variable	AUC (95% CI)	Sensitivity	Specificity	Youden index
Male				
BMI (kg/m^2^) + other factors	0.633 (0.621–0.644)	0.598	0.603	0.201
WC (cm) + other factors	0.639 (0.627–0.651)	0.591	0.616	0.207
WHtR + other factors	0.632 (0.620–0.643)	0.574	0.623	0.197
CUN-BAE + other factors	0.642 (0.630–0.653)	0.710	0.500	0.210
Female				
BMI (kg/m^2^) + other factors	0.608 (0.599–0.618)	0.634	0.534	0.168
WC (cm) + other factors	0.606 (0.596–0.615)	0.606	0.551	0.157
WHtR + other factors	0.607 (0.597–0.616)	0.627	0.528	0.156
CUN-BAE + other factors	0.614 (0.605–0.623)	0.651	0.523	0.175

*BMI* body mass index, *WC* waist circumference, *WHtR* waist-to-height ratio, *CUN-BAE* Clínica Universidad de Navarra-Body Adiposity Estimator.

## Discussion

In this cross-sectional study, we investigated the association between CUN-BAE and the prevalence of CM in a Chinese elderly population and compared the strength of the association between CUN-BAE and BMI, WC, and WHtR with CM. We found that increased CUN-BAE was associated with an increased prevalence of CM and that CUN-BAE was more strongly associated with the prevalence of CM than BMI, WC, and WHtR. The same results were found in the sex and age subgroup analysis. Dose–response relationships by restricted cubic spline analysis revealed a non-linear relationship between CUN-BAE, WC, WHtR, and CM in the total population. In addition, we found that CUN-BAE was a better predictor of CM compared to BMI, WC, and WHtR, both in men and women.

This is the first population-based cross-sectional study with a large sample size to examine the relationship between CUN-BAE, BMI, WC, WHtR, and the prevalence of CM and to determine the best predictors of CM. Due to the infeasibility of using expensive techniques to measure body composition at a large-scale population level, prior research has relied heavily on the use of anthropometric methods to examine the relationship between obesity and cardiometabolic disease^21–23^. Traditionally, BMI is the most widely used anthropometric index to define obesity, as its calculation requires only simple height and weight information. Although simple and reproducible, it has been criticized for its inherent weakness in distinguishing between fat and lean body mass^24^. In addition, the WC and WHtR, which are used to measure central obesity, have also been criticized because they do not take into account important factors related to adiposity, especially age, gender, or race^12,13^. In contrast, the anthropometric index CUN-BAE, calculated based on age, sex, and BMI, showed the highest correlation with direct measures of body fat and was considered a better indicator of body fat distribution^14^. Currently, limited studies are exploring the association of anthropometric indicators with CM. To our knowledge, only three studies have explored the association of obesity indicators with CM^4,5,11^. The study conducted by Kivimäki et al. involving 120,813 adults from the United States and Europe indicated that the risk of CM increased with increasing BMI^4^. However, this study only included European and American populations and did not include Asians, and our study provides new evidence in this regard. A cohort study by Archana Singh-Manoux et al. showed that the risk of developing CM from a single cardiometabolic disease was 1.19 times higher in overweight/obese(BMI ≥ 24 kg/m^2^) patients than in healthy individuals^5^. Another cohort study of 10,521 middle-aged and older adults showed that BMI, WC, and WHtR were positively associated with CM and that WC and WHtR were better predictors of CM than BMI^11^. Consistent with these studies, a strong positive association of BMI, WC, and WHtR with CM was also found in our study. Notably, in contrast to the results of this study, our study did not find that WC and WHtR were better predictors of CM than BMI in women, which may be due to differences in the study population.

Our study showed that anthropometric measures including CUN-BAE, BMI, WC, and WHtR were positively correlated with CM. The exact mechanism of this positive association remains to be elucidated, but low-grade chronic inflammation, insulin resistance, and ectopic fat deposition may be the main contributors. First, obesity causes low-grade chronic inflammation, which translates into cardiometabolic stress and increased myocardial load with deleterious hemodynamic consequences, which in turn cause cardiometabolic disease^25^. Second, the adipose tissue of obese individuals produces large amounts of bioactive mediators that lead to insulin resistance, which in turn affects apolipoprotein A1 (apoA-I) production or hepatic high-density lipoprotein (HDL) secretion and finally induces the development of metabolic syndrome^26^, and insulin resistance also impedes normal cardiac function by inhibiting metabolic pathways and overstimulating growth factors^27^. Third, obese individuals secrete excessive amounts of free fatty acids outside their fat storage tissues, which are transferred to ectopic sites such as the heart and vascular system, causing ectopic fat deposition and eventually leading to the development of cardiometabolic diseases^28^.

In this study, the positive association between CUN-BAE and CM was stronger than that of BMI, WC, and WHtR. Several previous studies support our findings to some extent. A study conducted in Spain by Veronica Davila-Batista et al. found that the CUN-BAE index was more strongly associated with cardiometabolic conditions, including diabetes, arterial hypertension, and metabolic syndrome (Mets), compared with BMI and WC, suggesting that CUN-BAE may be better than BMI in identifying individuals at risk for cardiometabolic disease^17^. Xintong Guo et al. found that the association between CUN-BAE and metabolic syndrome was stronger than BMI, WHtR, and other indicators in diabetic patients over 60 years of age^29^. In addition, Vicente Martín et al. found that CUN-BAE showed a positive association with hypertension and diabetes and presented a better gradient than BMI in a population of adults over 18 years of age^18^. Remarkably, in the subgroup analysis stratified by gender, we found that the correlation between CUN-BAE and CM was attenuated and not significantly better than BMI, WC, WHtR, and CM. A sex-stratified analysis of 9555 Iranian subjects by Fahimeh Haghighatdoost et al. showed similar associations of CUN-BAE and BMI with cardiovascular disease risk factors, including metabolic syndrome, hypercholesterolemia, and hypertension^30^. Paradoxically, a prospective cohort study of 6796 individuals in Norway showed that CUN-BAE was more strongly associated with hypertension, diabetes, angina, and stroke than BMI when analyzed stratified by sex, yet when men and women were combined in the analysis, the association of CUN-BAE with all outcomes dropped below BMI. The differences from our findings may reflect the different outcomes measured (cardiovascular events vs cardiometabolic multimorbidity). This may also be related to ethnic differences in body composition^31^ and the contribution of systemic obesity to the risk of CM^4^.

The ROC curves and AUC were used to compare the predictive power of CUN-BAE, BMI, WC, and WHtR for CM. In both men and women, the CM predictive power of CUN-BAE was stronger than that of BMI, WC, and WHtR. Similar to our findings, a cohort study that included 15,464 adults found that CUN-BAE was the best predictor of diabetes, compared to BMI and WC^32^. A case–control study in normoglycemic adults showed that CUN-BAE could be the first simple/effective screening tool to identify increased fat mass and increased metabolic risk in lean individuals^33^. In addition, another cross-sectional study of 418,343 individuals conducted in Spain found that CUN-BAE was superior to BMI, WC, and WHtR in identifying metabolic syndrome^34^. To our knowledge, only the study by Yanqiang Lu et al.^11^ explored the predictive power of anthropometric measures for CM, which found that WC was a better predictor of CM than BMI, which is consistent with our results in men. However, this study had a small sample size and did not exclude the effects of confounding factors such as physical activity, which is an important modifiable risk factor for cardiometabolic disease. Our study remedies these deficiencies. Our study supports for the first time the strong correlation between the CUN-BAE index and CM in Asian populations and suggests that CUN-BAE is a better predictor of CM than BMI, WC, and WHtR. CUN-BAE may be a better measure of the adverse effect of adiposity on the prevalence of CM than BMI, WC, and WHtR. More studies need to be conducted to further support our findings.

Our study has several strengths. First, we are the first study to explore the association between CUN-BAE indicators and CM in an Asian population, filling a gap in the study of CUN-BAE indicators in Asian populations. Second, the large sample size, the standardized measures used, and the use of an annual health examination dataset in this study avoided recall bias to some extent. Finally, the AUCs were used to compare the predictive power of anthropometric indicators CUN-BAE, BMI, WC, and WHtR for CM in older individuals, which could be of practical value to improve related studies. However, some limitations of this study should be noted. First, this study focused on the elderly population, so it was not possible to compare the relationship between obesity indicators and the prevalence of CM in other age groups, which limits the generalizability of this study. Second, this study is a cross-sectional study, so it is difficult to examine the causal relationship between exposure and outcome. Finally, although many confounding factors were adjusted for in the analysis of this study, there were still some potential confounding factors present that were not adjusted for, such as literacy and dietary habits.

## Conclusion

Our study found that increased CUN-BAE was associated with an increased prevalence of CM in the Chinese elderly population and that CUN-BAE was more strongly associated with the prevalence of CM than BMI, WC, and WHtR. The same results were found in the analysis of sex and age stratification. The predictive power of CUN-BAE for CM was better than that of BMI, WC, and WHtR. Our findings suggest that CUN-BAE may be a better measure of the adverse effect of adiposity on the prevalence of CM than BMI, WC, and WHtR.

### Supplementary Information

Supplementary Information.

## Data Availability

The data underlying this study are not publicly available because our data comes from the Xinzheng Health Commission, a third-party organization, and we have signed a data confidentiality agreement. However, such data can be accessed from the authors or non-author point of contact upon reasonable request. Data requests can be made to corresponding author Songhe Shi (ssh@zzu.edu.cn) or 62693484@163.com, an institutional, non-author point of contact.
