# Supplementary material for: Association of the CUN-BAE body adiposity estimator and other obesity indicators with cardiometabolic multimorbidity: a cross-sectional study
Source: Sci Rep. 2024 May 8;14:10557. doi: 10.1038/s41598-024-52164-7 (PMC11078937; doi:10.1038/s41598-024-52164-7)
Supplement: Supplementary file 1 — Supplementary Information. [file 41598_2024_52164_MOESM1_ESM.docx]

**
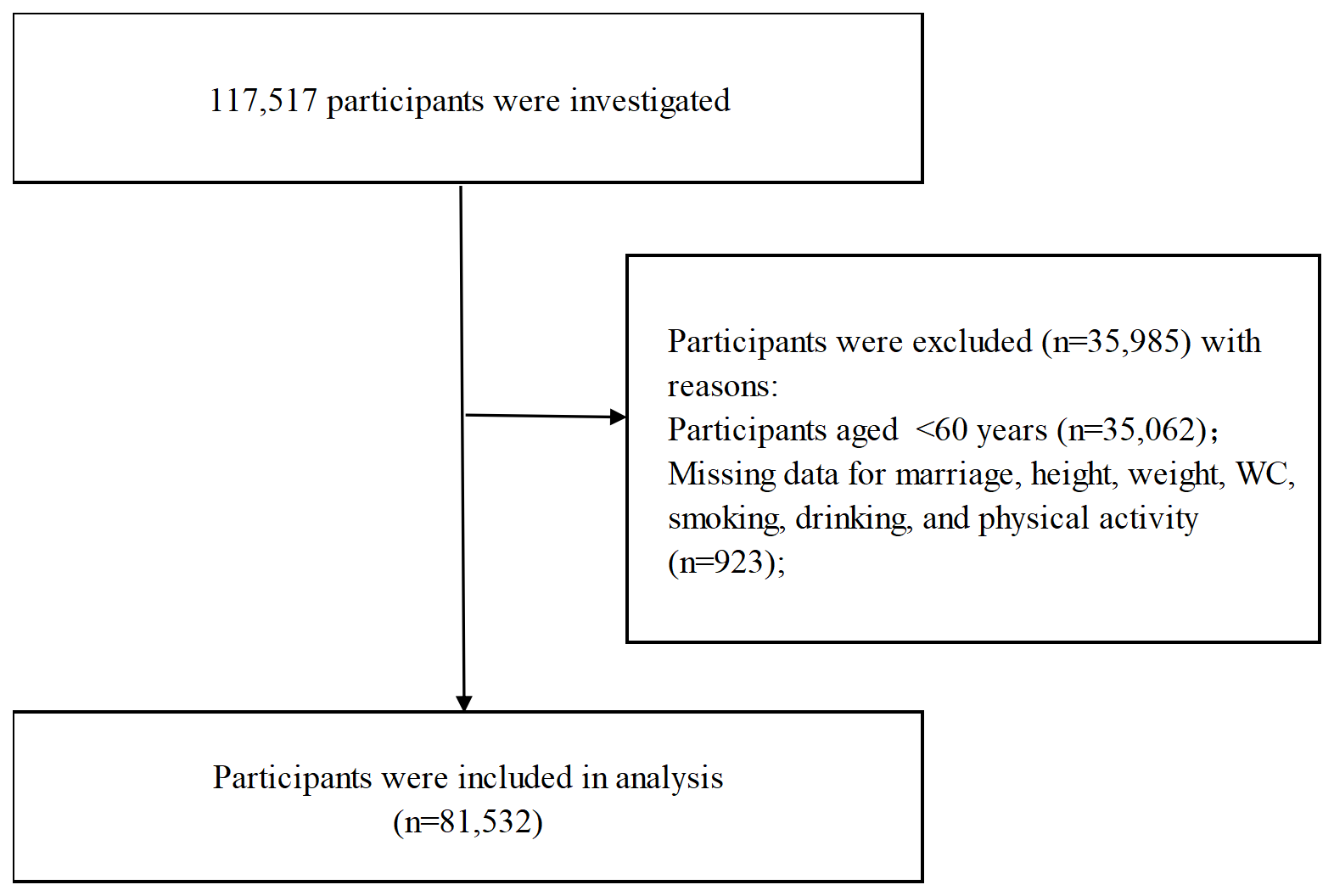
**

**Figure S1** Flow diagram of participant selection

| **Table S1 The Pearson correlations of all anthropometric indices** | | | | |
| --- | --- | --- | --- | --- |
| Indices | CUN-BAE | BMI | WC | WHtR |
| CUN-BAE | 1 | 0.582* | 0.281* | 0.560* |
| BMI | 0.582* | 1 | 0.683* | 0.684* |
| WC | 0.281* | 0.683* | 1 | 0.871* |
| WHtR | 0.560* | 0.684* | 0.871* | 1 |

CUN-BAE, Clínica Universidad de Navarra-Body Adiposity Estimator; BMI, body mass index; WC, waist circumference; WHtR, waist-to-height ratio.

*:P<0.001, statistically significant.

| **Table S2** **Association between BMI, WC, WHtR, CUN-BAE, and CM by age** | | | | |  |
| --- | --- | --- | --- | --- | --- |
| **Variables** | **No. of cases** | **Model 1**  **OR (95%CI)** | **Model 2**  **OR (95%CI)** | **Model 3**  **OR (95%CI)** |  |
| Younger elderly |  |  |  |  |  |
| BMI |  |  |  |  |  |
| <P^25^ | 636 | Reference | Reference | Reference |  |
| P^25^-P^50^ | 844 | 1.342 (1.208-1.491) | 1.363 (1.227-1.515) | 1.337 (1.02-1.486) |  |
| P^50^-P^75^ | 1,084 | 1.748 (1.581-1.933) | 1.738 (1.571-1.923) | 1.676 (1.514-1.856) |  |
| ≥P^75^ | 1,461 | 2.414 (2.193-2.657) | 2.335 (2.120-2.571) | 2.194 (1.989-2.419) |  |
| P trend |  | <0.001 | <0.001 | <0.001 |  |
| Continuous per SD | 4,025 | 1.373 (1.332-1.415) | 1.348 (1.308-1.389) | 1.316 (1.276-1.357) |  |
| WC |  |  |  |  |  |
| <P^25^ | 661 | Reference | Reference | Reference |  |
| P^25^-P^50^ | 728 | 1.105 (0.992-1.231) | 1.141 (1.024-1.271) | 1.108 (0.994-1.235) |  |
| P^50^-P^75^ | 1,046 | 1.437 (1.300-1.588) | 1.516 (1.371-1.677) | 1.442 (1.303-1.596) |  |
| ≥P^75^ | 1,590 | 2.065 (1.881-2.268) | 2.159 (1.965-2.372) | 2.013 (1.830-2.216) |  |
| P trend |  | <0.001 | <0.001 | <0.001 |  |
| Continuous per SD | 4,025 | 1.344 (1.304-1.385) | 1.359 (1.318-1.400) | 1.326 (1.286-1.368) |  |
| WHtR |  |  |  |  |  |
| <P^25^ | 498 | Reference | Reference | Reference |  |
| P^25^-P^50^ | 943 | 1.498 (1.340-1.674) | 1.445 (1.293-1.615) | 1.388 (1.241-1.552) |  |
| P^50^-P^75^ | 1,027 | 1.898 (1.701-2.117) | 1.762 (1.578-1.969) | 1.655 (1.480-1.850) |  |
| ≥P^75^ | 1,557 | 2.788 (2.513-3.092) | 2.458 (2.211-2.732) | 2.253 (2.024-2.508) |  |
| P trend |  | <0.001 | <0.001 | <0.001 |  |
| Continuous per SD | 4,025 | 1.402 (1.361-1.444) | 1.342 (1.301-1.383) | 1.304 (1.263-1.345) |  |
| CUN-BAE |  |  |  |  |  |
| <P^25^ | 550 | Reference | Reference | Reference |  |
| P^25^-P^50^ | 967 | 1.809 (1.625-2.013) | 1.866 (1.674-2.081) | 1.782 (1.597-1.988) |  |
| P^50^-P^75^ | 1,002 | 1.882 (1.692-2.093) | 2.790 (2.369-3.284) | 2.595 (2.201-3.059) |  |
| ≥P^75^ | 1,506 | 2.918 (2.639-3.225) | 4.372 (3.681-5.192) | 3.930 (3.302-4.678) |  |
| P trend |  | <0.001 | <0.001 | <0.001 |  |
| Continuous per SD | 4,025 | 1.446 (1.399-1.495) | 1.814 (1.710-1.925) | 1.733 (1.631-1.841) |  |
| Older adults |  |  |  |  |  |
| BMI |  |  |  |  |  |
| <P^25^ | 267 | Reference | Reference | Reference |  |
| P^25^-P^50^ | 374 | 1.432 (1.218-1.685) | 1.428 (1.213-1.680) | 1.437 (1.221-1.692) |  |
| P^50^-P^75^ | 490 | 1.923 (1.648-2.245) | 1.893 (1.621-2.212) | 1.904 (1.628-2.226) |  |
| ≥P^75^ | 611 | 2.466 (2.123-2.864) | 2.376 (2.042-2.764) | 2.377 (2.040-2.770) |  |
| P trend |  | <0.001 | <0.001 | <0.001 |  |
| Continuous per SD | 1,742 | 1.362 (1.300-1.428) | 1.339 (1.277-1.404) | 1.339 (1.276-1.405) |  |
| WC |  |  |  |  |  |
| <P^25^ | 246 | Reference | Reference | Reference |  |
| P^25^-P^50^ | 354 | 1.134 (0.959-1.342) | 1.161 (0.981-1.375) | 1.158 (0.978-1.372) |  |
| P^50^-P^75^ | 505 | 1.580 (1.349-1.851) | 1.630 (1.389-1.912) | 1.614 (1.375-1.895) |  |
| ≥P^75^ | 637 | 2.149 (1.843-2.506) | 2.217 (1.898-2.589) | 2.171 (1.856-2.541) |  |
| P trend |  | <0.001 | <0.001 | <0.001 |  |
| Continuous per SD | 1,742 | 1.349 (1.287-1.415) | 1.357 (1.293-1.423) | 1.348 (1.284-1.415) |  |
| WHtR |  |  |  |  |  |
| <P^25^ | 249 | Reference | Reference | Reference |  |
| P^25^-P^50^ | 365 | 1.166 (0.987-1.377) | 1.151 (0.974-1.360) | 1.136 (0.961-1.343) |  |
| P^50^-P^75^ | 423 | 1.455 (1.237-1.712) | 1.408 (1.195-1.659) | 1.376 (1.167-1.623) |  |
| ≥P^75^ | 705 | 2.091 (1.799-2.431) | 1.990 (1.706-2.322) | 1.924 (1.646-2.248) |  |
| P trend |  | <0.001 | <0.001 | <0.001 |  |
| Continuous per SD | 1,742 | 1.329 (1.268-1.393) | 1.308 (1.246-1.373) | 1.295 (1.232-1.360) |  |
| CUN-BAE |  |  |  |  |  |
| <P^25^ | 300 | Reference | Reference | Reference |  |
| P^25^-P^50^ | 452 | 1.559 (1.339-1.814) | 1.692 (1.445-1.981) | 1.670 (1.425-1.958) |  |
| P^50^-P^75^ | 393 | 1.338 (1.145-1.563) | 1.918 (1.483-2.480) | 1.898 (1.466-2.458) |  |
| ≥P^75^ | 597 | 2.129 (1.841-2.460) | 3.070 (2.370-3.977) | 3.027 (2.331-3.931) |  |
| P trend |  | <0.001 | <0.001 | <0.001 |  |
| Continuous per SD | 1,742 | 1.306 (1.242-1.374) | 1.775 (1.618-1.947) | 1.774 (1.615-1.949) |  |
| OR, odd ratio; CI, confdential interval; BMI, body mass index; WC, waist circumference; WHtR , waist-to-height ratio; CUN-BAE, Clínica Universidad de Navarra-Body Adiposity Estimator; SBP, systolic blood pressure; DBP, diastolic blood pressure; RHR resting heart rate.  Model 1: unadjusted.  Model 2: adjusted for age, sex, marital status.  Model 3: Model 2 plus smoking, drinking, physical activity, SBP, DBP, and RHR. | | | | |  |

**Table S3** **Tests of interaction for CUN-BAE, BMI, WC and WHtR for CM.**

|  |  | **CM** |
| --- | --- | --- |
| CUN-BAE | Sex | 0.006 |
|  | Age (60-74 and ≥75) | 0.057 |
| BMI | Sex | <0.001 |
|  | Age (60-74 and ≥75) | 0.951 |
| WC | Sex | <0.001 |
|  | Age (60-74 and ≥75) | 0.938 |
| WHtR | Sex | <0.001 |
|  | Age (60-74 and ≥75) | 0.250 |
| BMI, body mass index; WC, waist circumference; WHtR , waist-to-height ratio; CUN-BAE, Clínica Universidad de Navarra-Body Adiposity Estimator. | | |

| **Table S4** AUCs for anthropometric indices in relation to Cardiovascular disease | | | | | | |
| --- | --- | --- | --- | --- | --- | --- |
| **Variable** | **Diabetes** | | **Stroke** | | **Coronary heart disease** | |
|  | **AUC** | **(95%CI)** | **AUC** | **(95%CI)** | **AUC** | **(95%CI)** |
| Male |  |  |  |  |  |  |
| BMI (kg/m^2^) + other factors | 0.619 | (0.613-0.625) | 0.566 | (0.547-0.585) | 0.581 | (0.572-0.589) |
| WC (cm) + other factors | 0.618 | (0.612-0.624) | 0.565 | (0.546-0.584) | 0.590 | (0.582-0.598) |
| WHtR + other factors | 0.610 | (0.604-0.616) | 0.567 | (0.548-0.586) | 0.594 | (0.585-0.602) |
| BAE + other factors | 0.616 | (0.610-0.622) | 0.575 | (0.556-0.594) | 0.590 | (0.582-0.599) |
| Female |  |  |  |  |  |  |
| BMI (kg/m^2^) + other factors | 0.620 | (0.614-0.625) | 0.549 | (0.529-0.569) | 0.565 | (0.559-0.572) |
| WC (cm) + other factors | 0.619 | (0.61.3-0.624) | 0.547 | (0.527-0.567) | 0.562 | (0.556-0.569) |
| WHtR + other factors | 0.611 | (0.605-0.616) | 0.553 | (0.533-0.573) | 0.569 | (0.563-0.576) |
| BAE + other factors | 0.620 | (0.614-0.625) | 0.558 | (0.538-0.577) | 0.573 | (0.566-0.580) |
| BMI, body mass index; WC, waist circumference; WHtR, waist-to-height ratio; CUN-BAE, Clínica Universidad de Navarra-Body Adiposity Estimator. | | | | | | |
